# A2E Distribution in RPE Granules in Human Eyes

**DOI:** 10.3390/molecules25061413

**Published:** 2020-03-20

**Authors:** Ziqiang Guan, Yiwen Li, Shuliang Jiao, Nusrat Yeasmin, Philip J. Rosenfeld, Sander R. Dubovy, Byron L. Lam, Rong Wen

**Affiliations:** 1 Department of Biochemistry, Duke University Medical Center, Durham, NC 27710, USA; 2 Bascom Palmer Eye Institute, Miller School of Medicine, University of Miami, Miami, FL 33136, USA; yli2@med.miami.edu (Y.L.); prosenfeld@med.miami.edu (P.J.R.); sdubovy@med.miami.edu (S.R.D.); blam@med.miami.edu (B.L.L.); 3 Department of Biomedical Engineering, Florida International University, Miami, FL 33174, USA; shjiao@fiu.edu (S.J.); yeasminnusrat@gmail.com (N.Y.)

**Keywords:** A2E (*N-*retinylidene-*N-*retinylethanolamine), RPE lipofuscin, lipofuscin fluorescence, retina, liquid chromatography/mass spectrometry (LC/MS), melanin

## Abstract

A2E (*N-*retinylidene-*N-*retinylethanolamine) is a major fluorophore in the RPE (retinal pigment epithelium). To identify and characterize A2E-rich RPE lipofuscin, we fractionated RPE granules from human donor eyes into five fractions (F1–F5 in ascending order of density) by discontinuous sucrose density gradient centrifugation. The dry weight of each fraction was measured and A2E was quantified by liquid chromatography/mass spectrometry (LC/MS) using a synthetic A2E homolog as a standard. Autofluorescence emission was characterized by a customer-built spectro-fluorometer system. A significant A2E level was detected in every fraction, and the highest level was found in F1, a low-density fraction that makes up half of the total weight of all RPE granules, contains 67% of all A2E, and emits 75% of projected autofluorescence by all RPE granules. This group of RPE granules, not described previously, is therefore the most abundant RPE lipofuscin granule population. A progressive decrease in autofluorescence was observed from F2 to F4, whereas no autofluorescence emission was detected from the heavily pigmented F5. The identification of a novel and major RPE lipofuscin population could have significant implications in our understanding of A2E and lipofuscin in human RPE.

## 1. Introduction

Lipofuscin, a hallmark of aging [1], is formed in the process of a conserved catabolic mechanism known as autophagy [2,3] in which cellular components are digested in lysosomes and the undegradable material accumulates to form a polymorphous pigment waste, collectively called lipofuscin [1,4]. The molecular composition of lipofuscin is highly heterogeneous, depending on cell types and metabolic origins [5,6]. In phagocytes, such as macrophages and retinal pigment epithelium (RPE), lipofuscin is formed by undegradable waste from phagocytosed materials [1].

The RPE is a monolayer of pigmented cells serving multiple functions [7,8]. One important function is to phagocytose the shed outer segments of photoreceptors daily [9,10], which leads to a significant accumulation of lipofuscin [11]. It has long been suggested that lipofuscin in RPE plays a role in retinal diseases, including age-related macular degeneration (AMD) and Stargardt disease [6,11].

The first identified lipofuscin fluorophore in the RPE was an amphoteric quaternary amine [12]. Its structure was later determined to be a pyridinium bisretinoid, *N-*retinylidene-*N-*retinylethanolamine (A2E) [13]. A2E, a byproduct of the visual cycle, is a major RPE lipofuscin fluorophore [14,15]. It is believed that A2E affects the normal functions of the RPE and is involved in retinal diseases. Strategies designed to inhibit A2E formation for AMD and Stargardt disease by inhibiting key visual cycle enzymes or using deuterated vitamin A (C20-D_3_-Vitamin A), respectively, are in development [16,17,18,19]. Most notably, a phase 2 clinical trial using deuterated vitamin A for Stargardt disease is ongoing [20].

RPE lipofuscin has been purified by discontinuous sucrose gradient centrifugation. Boulton and colleagues identified RPE lipofuscin as a fraction at the interface between 1.2 and 1.4 M of sucrose [21,22]. Similar fractionation methods have been adopted by other investigators to isolate RPE lipofuscin granules for lipofuscin studies, including morphological, lipid, proteomic, and melanin oxidation analyses [23,24,25,26]. The lipofuscin fraction was originally identified by fluorescence microscopy. Questions remain as to whether the fraction identified by Boulton and colleagues and accepted by others is RPE lipofuscin, and whether other RPE lipofuscin exists.

We attempted to answer these questions by characterizing the A2E content in RPE granules. Our rationale was that A2E, as a major RPE lipofuscin fluorophore, should be rich in RPE lipofuscin. A2E therefore could be used as a biomarker for identification of RPE lipofuscin in fractionated RPE granules. In the present work, we systematically analyzed the distribution and quantities of A2E in human RPE granules and identified a novel major A2E-rich population of RPE granules. In addition, A2E was found in all RPE granules. Interestingly, the RPE lipofuscin, previously reported by Boulton and colleagues [21], was the least abundant group of RPE granules. These findings could change our current understanding of A2E and RPE lipofuscin.

## 2. Results

### 2.1. Morphological Analysis of Granules

**Figure 1:**
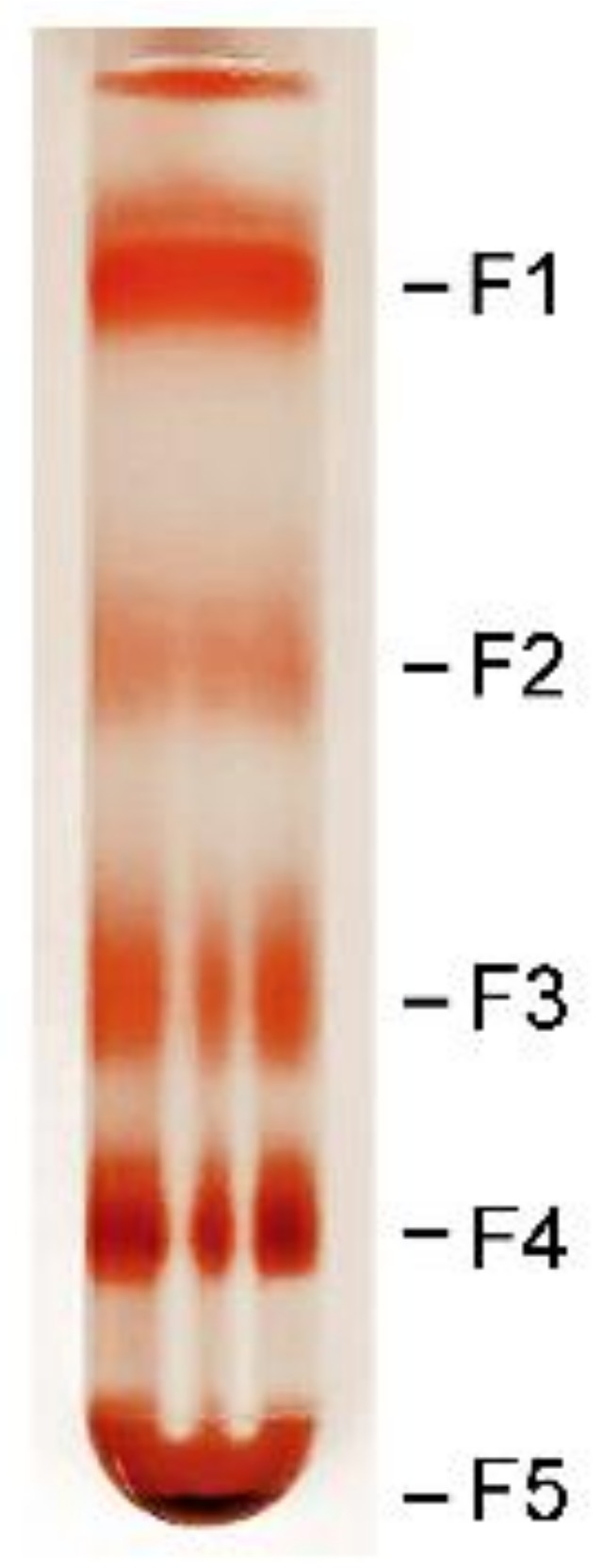
Fractionation of retinal pigment epithelium (RPE) granules by discontinuous sucrose density gradient centrifugation. Five bands (F1–F5) were clearly visible after centrifugation.

RPE cells from human donor eyes were lysed and granules were fractionated by discontinuous sucrose density gradient centrifugation with four sucrose concentrations (2, 1.4, 1.2, and 1.0 M). Four fractions were visible at the interfaces of 0–1.0, 1.0–1.2, 1.2–1.4, and 1.4–2.0 M sucrose, designated as Fraction 1 (F1), F2, F3, and F4, respectively. A pellet denser than 2.0 M sucrose, designated as F5, was formed at the bottom of the tube (Figure 1).

**Figure 2:**
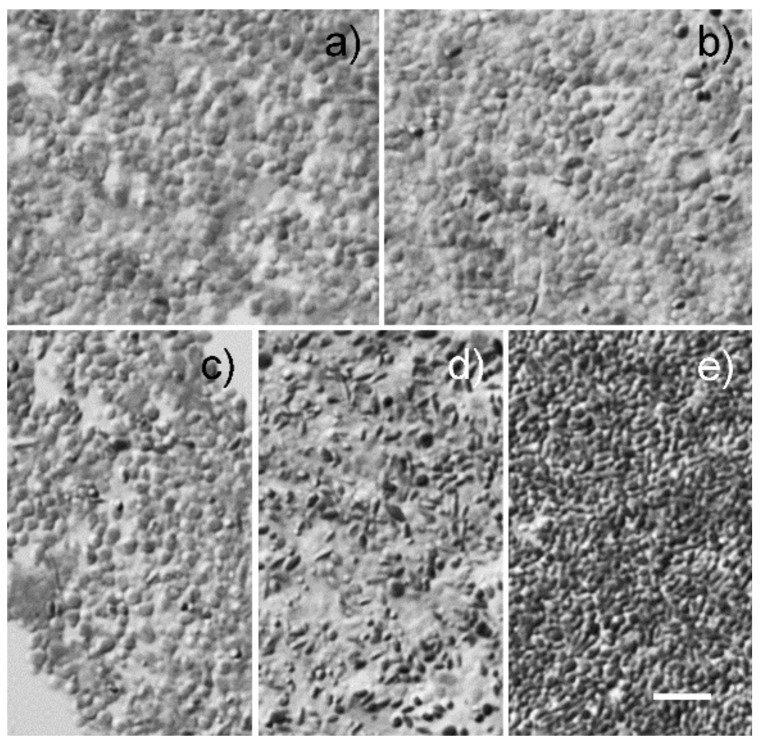
RPE granule morphology. Semi-thin sections of granules were examined by DIC (differential interference contrast) light microscopy without staining. Representative images of granules in F1, F2, F3, F4, and F5 are shown in panel **a**, **b**, **c**, **d**, and **e**, respectively. Scale bar: 10 µm.

Morphologically, granules in F1 are round particles of about 2–3 µm in size with no dark pigment (Figure 2a). In fraction F2, granules are similar in size to those in F1, but some contained dark pigment (Figure 2b). Granules in F3 are more heterogeneous in size with many small pigmented granules (Figure 2c). In F4, granules are generally smaller than those in F1, F2, and F3. Many cigar-shaped granules with dark pigment are present (Figure 2d). F5 granules are heavily pigmented and smaller than those in the other four fractions (Figure 2e).

### 2.2. Weight Distribution in Granule Fractions

**Figure 3:**
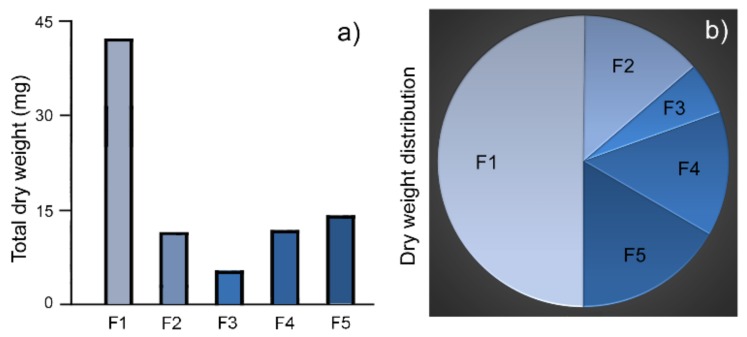
Dry weight of RPE granules. F1 is the most abundant population of RPE granules with the highest total dry weight (**a**). It also accounts for the largest proportion of the weight distribution in the 5 fractions of RPE granules (**b**).

The dry weight of granules in each fraction was measured in 100 µL suspension and the total weight of that fraction was calculated. The total dry weight of F1 is 42.3 mg, half (50.2%) of the combined weight of all RPE granules (84.2 mg, Figure 3a,b; Table 1). In comparison, the total dry weight of F3 is 4.9 mg, only 5.8% of the total granule weight of all fractions (Figure 3a,b; Table 1). By weight, the most abundant fraction F1 is 8.6-times as much as the least abundant fraction F3. The weights and distribution in the other three fractions were in the range of 11 to 14 mg, about 13% to 17% of the total weight of all five fractions (Figure 3; Table 1).

### 2.3. A2E Levels and Distribution in Granule Fractions

**Figure 4:**
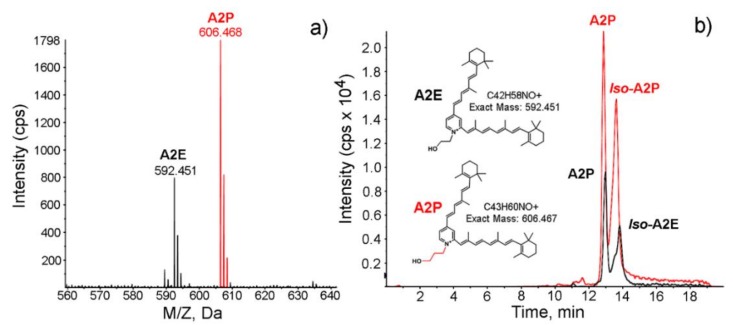
Quantification of A2E (*N-*retinylidene-*N-*retinylethanolamine) in lipofuscin granules by LC/MS. Panel (**a**) shows the positive ion ESI (electrospray ionization) mass spectrum of the M^+^ ions of A2E (in F1 granules, black peaks) and internal standard A2P (red peaks). Extracted ion chromatograms (EICs) of *m*/*z* 592.4 for A2E (black) and *m*/*z* 606.4 for A2P (red) are displayed in panel (**b**), along with the structures of A2E, A2P, and their exact masses.

The A2E level in 1 mg granules of each fraction was quantified by LC/MS with the synthesized A2P (*N-*retinylidene-*N-*retinylpropanolamine) as a spike-in internal standard. Figure 4a shows the positive mass spectrum of the M^+^ ions of A2E (in F1 granules, black peaks) and the spiked-in A2P (red peaks). The absolute amount of A2E in each fraction was quantified by comparing the peak area of the extracted ion chromatogram of A2E with that of the spiked-in synthetic A2P (Figure 4b).

**Figure 5:**
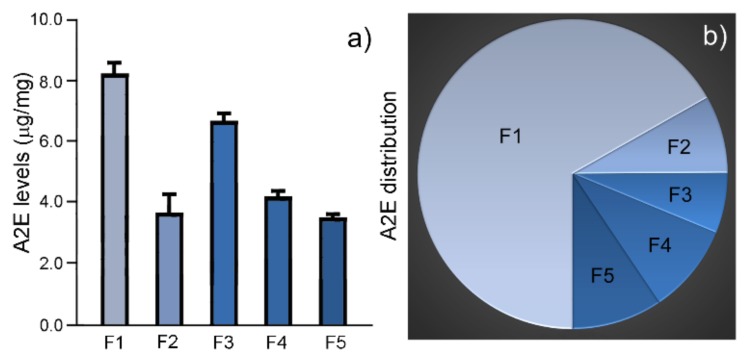
A2E levels and distribution. The A2E level in 1 mg of granules of each fraction was quantified by LC/MS. The highest A2E level was found in F1, followed by the level in F3 (**a**). F1 also accounts for the largest proportion of A2E distribution in the 5 fractions of RPE granules (**b**).

A significant A2E level was present in every fraction (Figure 5a; Table 2). The highest was found in F1 (8.3 ± 0.4 µg/mg, *n =* 3), followed by the level in F3 (6.7 ± 0.3 µg/mg, *n =* 3) (Figure 5a; Table 2). The levels in the other three fractions were around 4 µg/mg (Figure 5a; Table 2), about half of the level in F1.

The total A2E amount in a fraction was calculated by multiplying the total dry weight of the fraction by the amount of A2E measured in 1 mg granules. The A2E amount in F1 was 348.6 µg, 66.8% of the A2E in all five fractions (Figure 5b; Table 2). In contrast, the A2E amount in F3 was 32.9 µg, 6.3% of the A2E in all five fractions (Table 2). F1 contains 10-times more A2E than F3.

### 2.4. Fluorescence Spectroscopic Analysis

**Figure 6:**
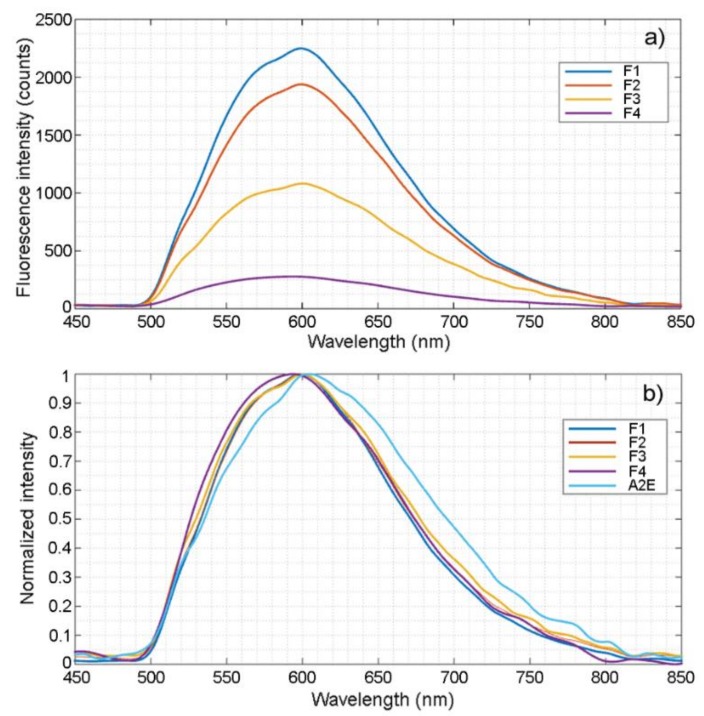
Autofluorescence emission by RPE granules. The emission spectrum of granules in each fraction (F1–F4) was in the range of 500 to 800 nm, peaked at 600 nm (**a**). Normalized spectral curves show that the curves are identical (**b**). The emission spectra from RPE granules are similar to that of synthetic A2E in H_2_O (**b**).

The autofluorescence emission from RPE granules was characterized using a custom-built spectro-fluorometer with high wavelength accuracy and responsivity. Granules from each fraction (1 mg in H_2_O) was placed in a quartz cuvette, excited by 488 nm laser light, and the fluorescence intensity and spectrum were measured. The emission spectrum of granules in each fraction (F1–F4) was in the range of 500 to 800 nm, peaked at 600 nm (Figure 6a). No fluorescence was detected in fraction F5 (not shown). When normalized, spectral curves from different fractions overlapped well (Figure 6b). The emission spectra from RPE granules were similar to that of synthetic A2E in H_2_O, with a slight (~5 nm) blue-shift (Figure 6b).

**Figure 7:**
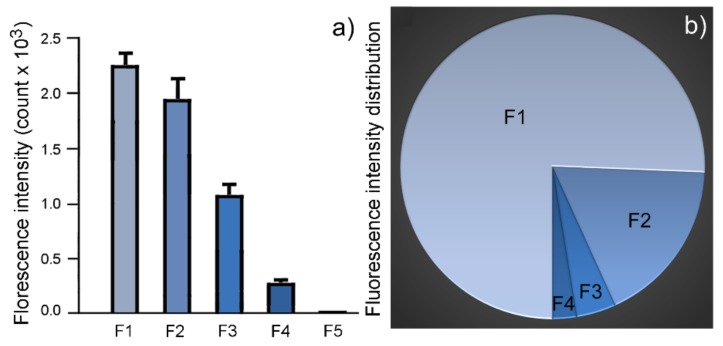
Fluorescence output from RPE granules. F1 had the highest fluorescence intensity counts per mg granules (**a**). A progressive decline of fluorescence intensity is seen from F2 to F4 (**a**). The projected fluorescence output from F1 is 75.3% of the total projected fluorescence output (**b**), (Table 3). Fraction F3 contributes only 4.2% to the total projected fluorescence output (**b**), (Table 3).

The intensity counts at 600 nm in each fraction (F1–F4) was taken as the representative fluorescence intensity of that fraction. F1 has the highest fluorescence output per mg of granules (2.247 × 10^3^ counts) (Figure 6a and Figure 7a; Table 3). A progressive decline in fluorescence intensity is observed from fraction F2 to fraction F4 (Figure 6a and Figure 7a; Table 3).

The total projected fluorescence emission from a fraction was calculated by multiplying the total weight of the fraction by the intensity counts measured from 1 mg granule of that fraction at 600 nm. The total projected fluorescence emission by F1 is 75.3% of the combined projected fluorescence output by all five fractions. The projected fluorescence output by F2 is 17.9% of the combined fluorescence output (Figure 7b; Table 3). Together, F1 and F2 contribute to more than 90% of the combined fluorescence output of all five fractions at 600 nm (Figure 7b; Table 3).

## 3. Discussion

Our systematic analysis of A2E in RPE granules yielded two major findings that could change our view of A2E and RPE lipofuscin in human eyes. One is the discovery of a low-density (<1.0 M sucrose) group of RPE granules (F1). This new group of granules is the most abundant RPE granules by weight, by A2E content, and by projected autofluorescence emission. When compared with F3, a fraction previously regarded as the RPE lipofuscin [21,24], the newly identified granule population is 8.6-times as much by weight, contains 10 times more A2E, and emits 18 times as much of projected autofluorescence output. The newly discovered granules population, having a high A2E level and fluorescence emission, should be regarded as lipofuscin granules. Thus, we have identified a major population of RPE lipofuscin granules that had not been reported previously.

The other major finding is the presence of a significant amount of A2E in every fraction of RPE granules, even in the heavily pigmented melanosomes. A2E was assumed to be at high level in the RPE lipofuscin granules but not in other granules. This view is no longer valid in light of the finding that A2E is widely distributed in RPE granules. Melanosomes are different from lipofuscin granules not only by their heavy pigmentation and higher density, but also by the lack of autofluorescence emission. And yet melanosomes (F5) contains 3.5 µg/mg A2E, almost half (42%) as much as the highest level of 8.3 µg/mg in F1. Our finding therefore raises a question as to how A2E, generated in photoreceptor OS and accumulated in the RPE in lipofuscin granules through phagocytosis of the shed OS tips [11], is transported to granules other than lipofuscin.

The total A2E from all 37 eyes measured in the present work is 521.7 µg or 14.1 µg/eye, much higher than the A2E amounts reported by previous studies. A2E was reported in the range of 200 to 800 ng per eye [27]. In another study, A2E was quantified as 7.8 × 10^−20^ mol per lipofuscin granule in the human RPE [28], leading to an estimation of 55 ng A2E per eye, given that a human eye has 3.56 million RPE cells [29] and that each cell contains 300 or so lipofuscin granules on average (in samples from individuals > 70-years-old) [30]. The significant difference in A2E levels between our study and those reported by others could be due to the different methodologies used. We quantified A2E by LC/MS, whereas others measured A2E with HPLC (high performance liquid chromatography) [27].

Our characterization of autofluorescence showed that F1 granules emitted the highest intensity count per gram (at 600 nm). The total projected autofluorescence emission by F1 was also the highest, accounting for 75% of the combined emission by all RPE granules. F1 granules are therefore the major source of fundus autofluorescence (FAF), a clinical test commonly used in retinal disease examinations [31,32]. In addition, we observed a progressive decrease in the autofluorescence output per gram of granules from F2 to F4. The decrease does not correlate with the A2E levels. Rather, it seemed to be inversely correlated with the pigmentation of RPE granules. It is likely that the decrease in autofluorescence output was due to the attenuating effect of the RPE pigment melanin, which absorbs both incident light and emitted fluorescence. The lack of autofluorescence output in F5 highlights the attenuating effect of melanin on autofluorescence, especially considering that the A2E level in F5 is comparable with that in F2 (Figure 5a; Table 2).

The fluorescence emission spectra from different fractions are identical, indicating that the fluorophores in those fractions were identical. The emission spectra of lipofuscin granules overlap well with the spectrum of synthetic A2E in H_2_O with a slightly blue-shifted (Figure 6b), which could be caused by other materials in the lipofuscin granules that might influence A2E fluorescence emission. For instance, a significant blue-shift of the emission spectrum (~30 nm) was observed when synthetic A2E was dissolved in methanol, as compared to A2E in H_2_O (S. Jiao, unpublished data).

The RPE cells used for the present work were collected from the entire eyes, not from specific regions. The eyes used in the present work were from old donors. Lipofuscin and A2E levels are known to increase with age so the distribution of A2E in younger eyes could be different.

The present work would not be possible without the 37 donor eyes from the Florida Lions Eye Bank, from which we extracted milligram quantities of RPE granules that allowed us to determine the dry weight and the relative abundance of each fraction, and to characterize the A2E content and autofluorescence output in equal amount (1 mg) of granules. In addition, we designed, synthesized A2P, and used it as a spike-in standard for quantifying the absolute amount of A2E by LC/MS. Furthermore, the emission spectra and the intensities autofluorescence emitted from RPE granules was characterized by a custom-built spectro-fluorometer with high wavelength accuracy and responsivity. These technical details ensured the accuracy and reliability of our measurements.

## 4. Materials and Methods

### 4.1. Isolation of Lipofuscin Granules From Human RPE Cells

RPE cells were isolated from 37 human donor eyes (ages ranging from 71 to 92 years of both genders with no known retinal diseases) obtained from Florida Lion Eye Bank (postmortem time: 2–7 days). To collect RPE cells, the anterior segment of an eye, including the cornea, the iris, and the lens, was dissected and discarded. The retina was gently and completely removed. RPE cells were detached from the Bruch membrane with a fine sable-hair brush in a small amount of phosphate-buffered saline (PBS). Cells collected were centrifuged at 30× g for 10 min at 4 °C, washed 3 times in PBS, and stored at −80 °C.

Cells collected were pooled, lysed by hypotonic shock with H_2_O, and homogenized in a Bullet Blender (Next Advance, Troy, NY, USA). RPE granules were fractionated by discontinuous sucrose density gradient centrifugation, modified from a published procedure [21]. To create the discontinuous sucrose density gradient, sucrose solutions of 4 concentrations, 2 M (2 mL), 1.4 M (2 mL), 1.2 M (3 mL), and 1.0 M (3 mL), were carefully added, layer by layer, into 14 × 89 mm centrifugation tubes. RPE homogenate (up to 2 mL/tube) was carefully layered onto the 1.0 M sucrose layer, and centrifuged in an SW41 rotor (Beckman, Indianapolis, IN, USA) at 25,000 rpm (107,170 *g*) for 1 h at 4 °C. Four layers, designated as F1 (Fraction 1), F2, F3, and F4, were formed at the interfaces between 0 and 1.0, 1.0 and 1.2, 1.2, and 1.4, and 1.4 and 2.0 M sucrose, respectively (Figure 1). A pellet (F5) denser than 2.0 M was formed at the bottom of the tube (Figure 1). Each fraction was carefully collected, washed 3 times with H_2_O, and pelleted by centrifuging in an SW41 rotor at 25,000 rpm for 30 min at 4 °C. Granules in each fraction was re-suspended in H_2_O before further analysis.

### 4.2. Dry Weight Measurement

The dry weight was measured in 100 µL of well-mixed granule suspension of a fraction. The sample was dried in a SpeedVac (Savant Instruments, Holbrook, NY, USA) overnight. The weight of a dried sample was measured in triplicate. The total dry weight of granules in a given fraction was calculated by multiplying the total volume of that fraction by the dry weight measured in 100 µL of the same fraction (Table 1).

### 4.3. Morphological Analysis

Granules of each fraction were fixed with 2.5% glutaraldehyde and 2% paraformaldehyde overnight, followed by 1% OsO_4_ for 1 h. Fixed samples were dehydrated, embedded in an Epon/Araldite mixture [33], sectioned at 1 µm thickness, and examined by DIC light microscopy without staining.

### 4.4. Synthesis of A2E and A2P

A2E was synthesized following a published method [34]. Ethanol (31.3 mL), ethanolamine (3.8 mL), acetic acid (4.5 mL), and all-trans-retinal (1 g) were added to a 50-mL tube and allowed to react at room temperature in the dark for 2 days with gentle rocking. The ethanol was then evaporated, and the reaction mixture was dissolved in acetonitrile, washed 5 times with hexane and 1 M sodium acetate (1:1). The middle layer was collected after each wash. The reaction product was washed one more time with H_2_O and dried in a SpeedVac (Savant Instruments) overnight. Synthesized A2E was stored under argon at −20 °C in the dark.

A2P, an A2E analog with an additional methylene group (14 D), was synthesized following the same method as A2E synthesis, except that propanolamine was used instead of ethanolamine. The two pyridinium bisretinoids have nearly identical ionization efficiencies in electrospray ionization/mass spectrometry (ESI/MS).

### 4.5. Quantification of A2E in RPE Granules

Total lipids were extracted from samples by a modified Bligh and Dyer method [35,36]. Briefly, 1 mg of RPE granules in suspension were mixed in 200 µL H_2_O with a Bullet Blender (Next Advance). Methanol (200 µL) was then added to the sample and mixed, followed by addition of 200 µL of chloroform (CHCl_3_) and mixing. The mixture was centrifuged at 14,000 rpm for 10 min in a tabletop microcentrifuge, and the lipid-containing lower phase was transferred to a collection tube. To ensure complete extraction, each sample was extracted 4 times with 200 µL fresh chloroform added each time. Collected lipids in chloroform were pooled and dried in a SpeedVac (Savant Instruments), flushed with argon, and stored at −20 °C in the dark until use.

The absolute amount of A2E in a sample was measured in triplicate by reverse-phase LC/MS. A known quantity of synthetic A2P standard was added to the total lipid extract from 1 mg of granules with final concentrations of 10 μg/mL for A2P and 1 mg/mL for RPE granules. This solution was further diluted 10-fold. Each LC/MS analysis used 5 μL of the final diluted solution. A Shimadzu LC system (comprising a solvent degasser, two LC−10A pumps, and an SCL−10A system controller) coupled to a Triple TOF5600 mass spectrometer (Sciex, Framingham, MA, USA) was operated at a flow rate of 200 μL/min with a linear gradient as follows: 100% of the mobile phase A was held isocratically for 2 min and then linearly increased to 100% mobile phase B over 14 min and held at 100% B for 4 min. Mobile phase A consisted of methanol/acetonitrile/aqueous 1 mM ammonium acetate (60/20/20, v/v/v). Mobile phase B consisted of 100% ethanol containing 1 mM ammonium acetate. A Zorbax SB-C8 reversed-phase column (5 μm, 2.1 × 50 mm) was obtained from Agilent (Palo Alto, CA, USA). The LC eluent was introduced into the ESI source of the mass spectrometer. Instrument settings for positive ion ESI/MS and MS/MS analysis of lipid species were as follows: Ion spray voltage (IS) = +5500 V; curtain gas (CUR) = 20 psi; ion source gas 1 (GS1) = 20 psi; de-clustering potential (DP) = +50 V; focusing potential (FP) = +150 V. Data acquisition and analysis were performed using the Analyst TF1.5 software (Sciex, Framingham, MA, USA).

### 4.6. Fluorescence Spectroscopy

The intensity and fluorescence spectrum of each sample (1 mg granules) was measured in quintuplicate with an epi-illumination spectro-fluorometer system built specifically for this work. A single-mode optical fiber-pigtailed 488 nm laser diode (LP488-SF20, Thorlabs, Newton, NJ, USA) was used as the excitation light source. The laser light was collimated after exiting the optical fiber, reflected by a dichroic mirror (DMLP505 long pass, 505 nm cutoff, Thorlabs), and focused onto the sample by a microscope objective lens (10×, 0.25 NA, EFL = 16.5 mm, NA = 0.13, Newport, Andover, MA, USA). The emitted fluorescence light was collected by the objective lens, passed through the dichroic mirror and a 488 nm notch filter (NF488–15, Thorlabs), then coupled into a multi-mode optical fiber, and detected by a spectrometer (USB4000, Ocean Optics, Largo, FL, USA). Wavelength accuracy and responsivity of the spectrometer were calibrated using a Xenon lamp in the 430–800 nm spectral range (6032, Newport) and a quartz Tungsten-Halogen lamp, respectively. A correction curve was established through the calibration procedure. Standard samples of fluorescein and rhodamine B were measured to confirm the spectral accuracy of the system.

## 5. Conclusions

We have identified a new and major lipofuscin granule population (F1) in human RPE cells. These granules are A2E-rich and are a major source of RPE autofluorescence. In addition, A2E is found in all RPE granules, including melanosomes, which raises a question as to how A2E is transported from lipofuscin to other RPE granules. Our autofluorescence data highlight the signal attenuating effects of melanin on RPE autofluorescence. The present work and described methodologies may have significant implications in our understanding of A2E and lipofuscin in the RPE.

## Figures and Tables

**Table 1:** Dry weights and distribution.

Fraction	Dry Weight (mg/100µL) (Mean ± SD)	Total Volume (µL)	Total Weight (mg)	Weight Distribution
F1	5.0 ± 0.0 (*n* = 3)	845	42.3	50.2%
F2	4.0 ± 0.0 (*n* = 3)	285	11.4	13.6%
F3	2.0 ± 0.0 (*n* = 3)	245	4.9	5.8%
F4	2.5 ± 0.0 (*n* = 3)	465	11.6	13.8%
F5	4.0 ± 0.1 (*n* = 3)	350	14.0	16.6%
Total		2190	84.2	100%

**Table 2:** A2E levels and distribution.

Fraction	A2E Level (µg/mg, Mean ± SD)	Total A2E	A2E Distribution
F1	8.3 ± 0.4 (*n =* 3)	348.6	66.8%
F2	3.7 ± 0.6 (*n =* 3)	42.2	8.1%
F3	6.7 ± 0.3 (*n =* 3)	32.9	6.3%
F4	4.2 ± 0.3 (*n =* 3)	48.8	9.4%
F5	3.5 ± 0.1 (*n =* 3)	49.3	9.4%
Total		521.7	100%

**Table 3:** A2E contents and distribution.

Fraction	Peak Intensity (at 600 nm, 1 mg in H_2_O) (Mean ± SD)	Projected Intensity	Projected Intensity Distribution
F1	2247 ± 126 (*n =* 5)	95,048	75.3%
F2	1983 ± 159 (*n =* 5)	22,606	17.9%
F3	1081 ± 86 (*n =* 5)	5297	4.2%
F4	273 ± 13 (*n =* 5)	3202	2.5%
F5	0 ± 0 (*n =* 5)	0	0%
Total		126,153	100%

## References

[1] Brunk U.T., Terman A. (2002). Lipofuscin: Mechanisms of age-related accumulation and influence on cell function. Free Radic. Biol. Med..

[2] Kaur J., Debnath J. (2015). Autophagy at the crossroads of catabolism and anabolism. Nat. Rev. Mol. Cell Biol..

[3] Dikic I., Elazar Z. (2018). Mechanism and medical implications of mammalian autophagy. Nat. Rev. Mol. Cell Biol..

[4] Sulzer D., Mosharov E., Talloczy Z., Zucca F.A., Simon J.D., Zecca L. (2008). Neuronal pigmented autophagic vacuoles: Lipofuscin, neuromelanin, and ceroid as macroautophagic responses during aging and disease. J. Neurochem..

[5] Katz M.L., Robison W.G. (2002). What is lipofuscin? Defining characteristics and differentiation from other autofluorescent lysosomal storage bodies. Arch. Gerontol. Geriatr..

[6] Moreno-Garcia A., Kun A., Calero O., Medina M., Calero M. (2018). An Overview of the Role of Lipofuscin in Age-Related Neurodegeneration. Front. Neurosci..

[7] Strauss O. (2005). The retinal pigment epithelium in visual function. Physiol. Rev..

[8] Strauss O., Kolb H., Nelson R., Fernandez E., Jones B. (2012). The retinal pigment epithelium. Webvision The Orignaization of the Retina and Visual System.

[9] Young R.W. (1967). The renewal of photoreceptor cell outer segments. J. Cell Biol..

[10] Young R.W., Bok D. (1969). Participation of the retinal pigment epithelium in the rod outer segment renewal process. J. Cell Biol..

[11] Sparrow J.R., Boulton M. (2005). RPE lipofuscin and its role in retinal pathobiology. Exp. Eye Res..

[12] Eldred G.E., Lasky M.R. (1993). Retinal age pigments generated by self-assembling lysosomotropic detergents. Nature.

[13] Sakai N., Decatur J., Nakanishi K., Eldred G.E. (1996). Ocular age pigment ‘‘A2-E’’: An unprecedented pyridinium bisretinoid. J. Am. Chem. Soc..

[14] Sparrow J.R., Fishkin N., Zhou J., Cai B., Jang Y.P., Krane S., Itagaki Y., Nakanishi K. (2003). A2E, a byproduct of the visual cycle. Vision Res..

[15] Ben-Shabat S., Parish C.A., Vollmer H.R., Itagaki Y., Fishkin N., Nakanishi K., Sparrow J.R. (2002). Biosynthetic studies of A2E, a major fluorophore of retinal pigment epithelial lipofuscin. J. Biol. Chem..

[16] Petrukhin K. (2013). Pharmacological inhibition of lipofuscin accumulation in the retina as a therapeutic strategy for dry AMD treatment. Drug. Discov. Today Ther. Strateg..

[17] Kaufman Y., Ma L., Washington I. (2011). Deuterium enrichment of vitamin A at the C20 position slows the formation of detrimental vitamin A dimers in wild-type rodents. J. Biol. Chem..

[18] Ma L., Kaufman Y., Zhang J., Washington I. (2011). C20-D3-vitamin A slows lipofuscin accumulation and electrophysiological retinal degeneration in a mouse model of Stargardt disease. J. Biol. Chem..

[19] Charbel Issa P., Barnard A.R., Herrmann P., Washington I., MacLaren R.E. (2015). Rescue of the Stargardt phenotype in Abca4 knockout mice through inhibition of vitamin A dimerization. Proc. Natl. Acad. Sci. USA.

[20] ClinicalTrials.gov Phase 2 Tolerability and Effects of ALK-001 on Stargardt Disease (TEASE). https://clinicaltrials.gov/ct2/show/NCT02402660.

[21] Boulton M., Docchio F., Dayhaw-Barker P., Ramponi R., Cubeddu R. (1990). Age-related changes in the morphology, absorption and fluorescence of melanosomes and lipofuscin granules of the retinal pigment epithelium. Vision Res..

[22] Boulton M., Marshall J. (1985). Repigmentation of human retinal pigment epithelial cells in vitro. Exp. Eye Res..

[23] Bazan H.E., Bazan N.G., Feeney-Burns L., Berman E.R. (1990). Lipids in human lipofuscin-enriched subcellular fractions of two age populations. Comparison with rod outer segments and neural retina. Investig. Ophthalmol. Visual Sci..

[24] Hong L., Garguilo J., Anzaldi L., Edwards G.S., Nemanich R.J., Simon J.D. (2006). Age-dependent photoionization thresholds of melanosomes and lipofuscin isolated from human retinal pigment epithelium cells. Photochem. Photobiol..

[25] Schutt F., Ueberle B., Schnolzer M., Holz F.G., Kopitz J. (2002). Proteome analysis of lipofuscin in human retinal pigment epithelial cells. FEBS Lett..

[26] Taubitz T., Fang Y., Biesemeier A., Julien-Schraermeyer S., Schraermeyer U. (2019). Age, lipofuscin and melanin oxidation affect fundus near-infrared autofluorescence. EBioMedicine.

[27] Parish C.A., Hashimoto M., Nakanishi K., Dillon J., Sparrow J. (1998). Isolation and one-step preparation of A2E and iso-A2E, fluorophores from human retinal pigment epithelium. Proc. Natl. Acad. Sci. USA.

[28] Davies S., Elliott M.H., Floor E., Truscott T.G., Zareba M., Sarna T., Shamsi F.A., Boulton M.E. (2001). Photocytotoxicity of lipofuscin in human retinal pigment epithelial cells. Free Radic. Biol. Med..

[29] Panda-Jonas S., Jonas J.B., Jakobczyk-Zmija M. (1996). Retinal pigment epithelial cell count, distribution, and correlations in normal human eyes. Am. J. Ophthalmol..

[30] Pollreisz A., Messinger J.D., Sloan K.R., Mittermueller T.J., Weinhandl A.S., Benson E.K., Kidd G.J., Schmidt-Erfurth U., Curcio C.A. (2018). Visualizing melanosomes, lipofuscin, and melanolipofuscin in human retinal pigment epithelium using serial block face scanning electron microscopy. Exp. Eye Res..

[31] Delori F.C., Dorey C.K., Staurenghi G., Arend O., Goger D.G., Weiter J.J. (1995). In vivo fluorescence of the ocular fundus exhibits retinal pigment epithelium lipofuscin characteristics. Investig. Ophthalmol. Visual Sci..

[32] Delori F., Keilhauer C., Sparrow J.R., Staurenghi G., Holz F., Schmitz-Valckenberg S., Spaide R., Bird A. (2007). Origin of fundus autofluorescence. Atlas of Fundus Autofluorescence Imaging.

[33] Lu J., Luo L., Huang D., Liu X., Xia X., Wang Z., Lam B.L., Yi J., Wen R., Li Y. (2018). Photoreceptor Protection by Mesencephalic Astrocyte-Derived Neurotrophic Factor (MANF). eNeuro.

[34] Penn J., Mihai D.M., Washington I. (2015). Morphological and physiological retinal degeneration induced by intravenous delivery of vitamin A dimers in rabbits. Dis. Model. Mech..

[35] Bligh E.G., Dyer W.J. (1959). A rapid method of total lipid extraction and purification. Can. J. Biochem. Physiol..

[36] Wen R., Lam B.L., Guan Z. (2013). Aberrant dolichol chain lengths as biomarkers for retinitis pigmentosa caused by impaired dolichol biosynthesis. J. Lipid Res..

